# Low-Dose Chest CT Protocols for Imaging COVID-19 Pneumonia: Technique Parameters and Radiation Dose

**DOI:** 10.3390/life13040992

**Published:** 2023-04-12

**Authors:** Ibrahim I. Suliman, Ghada A. Khouqeer, Nada A. Ahmed, Mohamed M. Abuzaid, Abdelmoneim Sulieman

**Affiliations:** 1 Department of Physics, College of Science, Imam Mohammad Ibn Saud Islamic University (IMSIU), Riyadh 11642, Saudi Arabia; 2 Deanship of Scientific Research, Imam Mohammad Ibn Saud Islamic University (IMSIU), Riyadh 11642, Saudi Arabia; 3 Faculty of Science, Taibah University, Al Madinah Al Munawwarah 42353, Saudi Arabia; 4 Medical Diagnostic Imaging Department, College of Health Sciences, University of Sharjah, Sharjah 27272, United Arab Emirates; 5 Radiology and Medical Imaging Department, College of Applied Medical Sciences, Prince Sattam Bin Abdulaziz University, Alkharj 11942, Saudi Arabia

**Keywords:** X-ray computed tomography, iterative reconstruction (IR), tube current modulation (TCM), spectrum-shaping tin filters, radiation dose, image quality

## Abstract

Chest computed tomography (CT) plays a vital role in the early diagnosis, treatment, and follow-up of COVID-19 pneumonia during the pandemic. However, this raises concerns about excessive exposure to ionizing radiation. This study aimed to survey radiation doses in low-dose chest CT (LDCT) and ultra-low-dose chest CT (ULD) protocols used for imaging COVID-19 pneumonia relative to standard CT (STD) protocols so that the best possible practice and dose reduction techniques could be recommended. A total of 564 articles were identified by searching major scientific databases, including ISI Web of Science, Scopus, and PubMed. After evaluating the content and applying the inclusion criteria to technical factors and radiation dose metrics relevant to the LDCT protocols used for imaging COVID-19 patients, data from ten articles were extracted and analyzed. Technique factors that affect the application of LDCT and ULD are discussed, including tube current (mA), peak tube voltage (kVp), pitch factor, and iterative reconstruction (IR) algorithms. The CTDIvol values for the STD, LDCT, and ULD chest CT protocols ranged from 2.79–13.2 mGy, 0.90–4.40 mGy, and 0.20–0.28 mGy, respectively. The effective dose (ED) values for STD, LDCT, and ULD chest CT protocols ranged from 1.66–6.60 mSv, 0.50–0.80 mGy, and 0.39–0.64 mSv, respectively. Compared with the standard (STD), LDCT reduced the dose reduction by a factor of 2–4, whereas ULD reduced the dose reduction by a factor of 8–13. These dose reductions were achieved by applying scan parameters and techniques such as iterative reconstructions, ultra-long pitches, and fast spectral shaping with a tin filter. Using LDCT, the cumulative radiation dose of serial CT examinations during the acute period of COVID-19 may have been inferior or equivalent to that of conventional CT.

## 1. Introduction

Chest X-ray radiography (CXR) and computed tomography (CT) are essential tools for the diagnosis and follow-up of COVID-19 pneumonia [1]. COVID-19 is a type of pneumonia that was first identified in Wuhan, Hubei Province, China, in 2019 [2,3]. Affected patients present with fever and cough in addition to non-specific symptoms, including fatigue, dyspnea, muscle soreness, and headache [4]. Timely detection, quick intervention, and appropriate health interventions can prevent the rapid spread of COVID-19 and reduce morbidity and mortality [5].

COVID-19 pneumonia can be diagnosed using different techniques, including real-time reverse transcription-polymerase chain reaction (RT-PCR), CXR, and CT. RT-PCR is the standard method for the diagnosis of COVID-19, while high-resolution chest CT is essential for the detection, diagnosis, and follow-up of the disease [6,7,8]. However, RT-PCR has several drawbacks, including a lack of test kits, a delay in obtaining results, and a low sensitivity of 60–70% when compared with chest CT and X-ray [9,10]. Similarly, chest X-rays demonstrate insufficient sensitivity when detecting ground-glass opacities (GGOs) and thus cannot detect the early stages of COVID-19 when compared to RT-PCR. Furthermore, chest radiography is associated with a high false-negative rate and poor sensitivity for detecting viral pneumonia [11,12].

Chest CT is a rapid and effective imaging tool for detecting COVID-19 pneumonia with a sensitivity of up to 95%, given the presence of classic CT findings [13]. Chest CT is more effective in detecting COVID-19 pneumonia, particularly after negative RT-PCR results [14,15]. Moreover, a CT scan can show a patient’s disease course and severity and localize the disease and its extent [16,17,18]. Hence, given their availability, chest CTs can prove useful for COVID-19 detection. Therefore, some organizations have recommended the use of chest CT for imaging COVID-19 [19]. However, the American College of Radiology and the Royal College of Radiologists in the UK, along with several other international organizations and professional bodies, have expressed concerns about the use of ionizing radiation for screening suspected or following up on COVID-19 pneumonia [20,21].

Chest CT is ideal for diagnosing pulmonary diseases. It provides an accurate and convenient way to identify pulmonary lesions and reveal their location, size, and other characteristics [22]. However, considering the increase in the population’s radiation burden from CT procedures, it is imperative to reduce unnecessary radiation exposure during these examinations [23]. COVID-19 patients can undergo an average of 6–8 CT scans during hospitalization, which can result in a cumulative effective dose of nearly 20 mSv [7,24]. However, chest CT imaging is challenging because of lung movement during breathing, which causes motion artefacts and increases image noise, thereby resulting in an overall degradation in image quality [24]. Furthermore, there is a paramount need for radiation dose reduction during chest CT scanning because it involves irradiation of radiosensitive organs, such as the breast and lungs [25,26].

Several authors have reviewed methods for achieving dose reduction during chest CT scans [27]. Furthermore, some studies have proposed low-dose CT (LDCT) and ultra-low-dose (ULD) chest CT protocols for imaging specific groups of populations [28,29]. As reported, the LDCT chest CT protocol provides an effective dose (ED) of approximately 1.5 mSv, while the ULDCT chest CT protocol provides an ED of approximately 0.5 mSv [30,31]. Despite the lack of a strict definition for the ULD chest CT protocol, the associated patient dose was close to that of the corresponding radiographic examinations.

Low-dose chest CT (LDCT and ULD) has several advantages for screening populations with high-risk diseases such as tuberculosis and COVID-19 pneumonia. In addition to significantly reducing the radiation exposure for patients, lung diseases can be identified more effectively using conventional chest radiography. Lowering the radiation dose reduces the likelihood of damage to the CT tubes and detectors, resulting in reduced operating costs and extended tube and detector lives.

Both the LDCT and ULD chest CT protocols are indispensable tools in the fight against COVID-19. Various authors who reported significant findings have reviewed these protocols [32,33]. However, studies comparing STD to LDCT or ULD are lacking.

Herein, we aimed to review the literature on COVID-19 chest CT protocols to outline actionable points to assist with LDCT and ULD protocol guidelines for COVID-19 imaging.

## 2. Materials and Methods

### 2.1. Search Strategy and Selection Criteria

This literature review aimed to address the differences in cumulative radiation exposure following multiple rounds of ionizing radiation used in different chest CT protocols for imaging COVID-19 pneumonia. Data for this literature review were collected through searches of various scientific databases, including the ISI Web of Science, Scopus, and PubMed, along with citations crawled from relevant articles. Combinations of the following keywords were used for the article search: X-rays/radiation dose/chest CT/COVID-19. We searched all literature published in 2019 and later.

### 2.2. Inclusion and Exclusion Criteria

The primary inclusion criteria were reports on technical parameters and radiation dose metrics relevant to chest CT use for both the diagnosis of suspected COVID-19 patients and the monitoring and follow-up of already diagnosed patients.

Literature reviews and internal reports, literature published in languages other than English, and studies that only reported the diagnostic performance of LDCT without reporting the radiation dose parameters were excluded.

### 2.3. Data Extraction

Radiation dose quantities such as the volume CT dose index (CTDIvol), the CT dose-length product (DLP), and other scanning parameters that were essential to the LDCT protocol were extracted from the reviewed studies. During the COVID-19 pandemic, the extracted data covered the phantom and patient dose surveys during chest CT scanning.

Two authors checked all the extracted data against the publication to ensure the completeness and accuracy of the collected data.

### 2.4. Radiation Dose Quantities in CT

Three types of CT dose quantities were used to report chest CT COVID-19 radiation dose results: *CTDI_vol_,* DLP, and ED [34,35,36]. The *CTDI_vol_* conveys information regarding the average dose absorbed in the scanned region, whereas the DLP reflects the total integrated absorbed dose for a complete CT examination. Finally, ED is a quantity that provides the idea of weighted radiation risk.

Primarily, the CT dose is modeled in terms of the CT dose index ($CTDI_{100}$), which is measured at the center ($CTDI_{100,c}$) and periphery ($CTDI_{100,p}$) of a standard head or body CT dosimetry phantom using a pencil ionization chamber with an active length of 100 mm. The weighted CT dose index was computed as shown in Equation (1):

(1) $$CTDI_{w}=\left(1/3\right)\cdot CTDI_{100,c}+\left(2/3\right)\cdot CTDI_{100,p}$$

*CTDI_vol_* considers the helical pitch or axial scan spacing and, hence, is related to *CTDI_w_* via:

(2) $$CTDI_{vol}=\left(1/p\right)\cdot CTDI_{w}$$

where p is the CT pitch factor, which was calculated using the formula: p=l/NT. where *N* is the number of simultaneously acquired tomographic slices, *T* is the slice thickness, and l is the couch movement per helical rotation. The overall energy delivered by a given scan protocol is better represented using the CT air kerma-length product, as shown in Equation (2) [34,35,36].

(3) $$DLP=CTDI_{vol}\cdot L$$

where *L* is the scan length. In the reviewed literature, the *CTDI_vol_* and *DLP* values in most centers were extracted from the DICOM header.

The effective dose is used to express the radiation risk for partial-body irradiation. It is defined as the sum of the dose absorbed by each of the specified body organs and tissues multiplied by the tissue-weighting factor for the same organ or tissue [8]:

(4) $$ED=\sum_{T}W_{T}\frac{H_{T}^{male}-H_{T}^{female}}{2}$$

**Figure 1:**
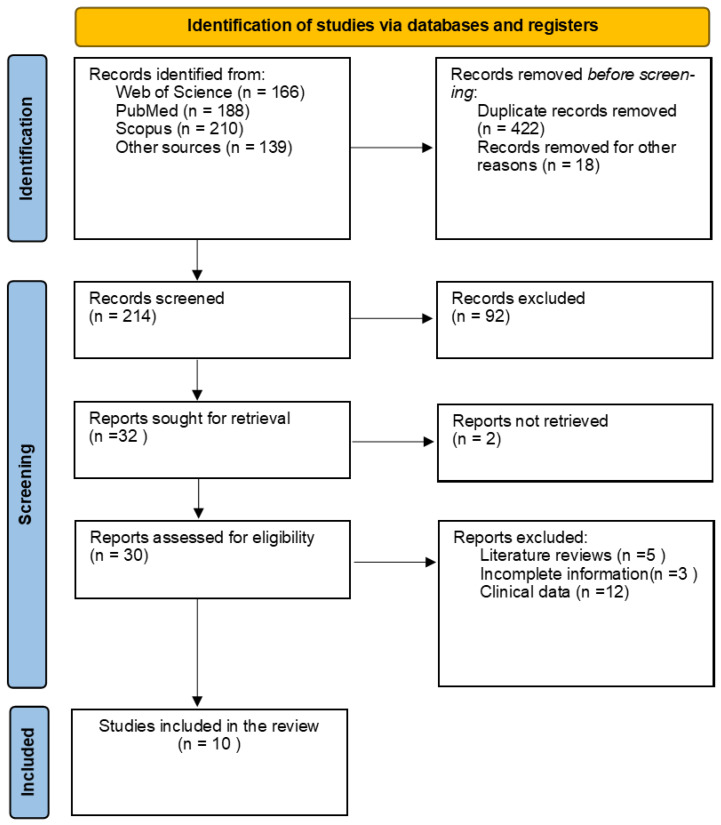
PRISMA flowchart showing the article search, inclusion, and exclusion processes.

*ED* refers to the average effective dose over age and sex, *W_T_* is the tissue weighting factor, and *H_T_* is the dose for organ *T*. In this literature survey, *ED* values were estimated by multiplying the *DLP* value by a conversion factor for chest CT equal to 100 kV:0.014-mSv/mGy·cm [37]. CT dosimetry software such as CT Expo and automatic dose registry software have also been used for effective dose calculations [38,39]. For multiple CT examinations, the cumulative ED can be obtained by summing the observed EDs of radiation received during hospitalization per patient undergoing the CT protocols. Figure 1 is a PRISMA flowchart showing the article search, inclusion, and exclusion processes.

## 3. Results and Discussion

### 3.1. Summaries of the Major Findings

Table 1 summarizes the surveyed studies, detailing the objectives of each study, the subjects and equipment used, and the major findings. The survey results are analyzed in the following subsections:

### 3.2. Scan Parameters and Techniques

Different strategies have been implemented for dose reduction in chest CT, including the lowering of kVp and mAs as well as the use of modulation along with the use of an ultra-long pitch factor, without adversely affecting the quality of diagnostic information. Table 2 presents the features of the LDCT, ULD, and STD chest CT protocols for imaging COVID-19 pneumonia. Based on the literature surveyed, these factors are discussed below.

Tube current (mA): Reducing the tube current is the first and most efficient way to reduce the radiation dose [40,41,42,43,44,45,46,47,48,49]. This is primarily achieved using tube current modulation (TCM), which adjusts the tube current according to a patient’s body characteristics. Survey studies have used either TCM or vendor-specific mA modulation software to achieve a dose reduction in up to 50% in standard-sized patients [50], including CareDose 4D in the mA modulation software from Siemens. The use of special mA modulation programs has helped professionals achieve true LDCT for imaging COVID-19.

Peak tube voltage (kVp): LDCT has been performed using tube voltages of less than or equal to 100 kVp [51]. Therefore, 80–100 kVp is commonly used in LDCT. Reducing the voltage from 120 kVp to 80 kVp increases the image noise and would require a four-fold increase in the tube current to maintain the image quality [52,53,54]. For better CT performance, spectral shaping with a tin filter at 100 kVp (100 Sn kVp) was used, which reduced low-energy X-rays, resulting in a significant dose reduction [55,56]. Another technique that can be used is dual-energy CT (DECT) for the LDCT protocol, which is centered on the concurrent acquisition of low (80 kVp) and high (140 kVp) energy X-rays [57]. Similarly, Agostini et al. [40] used high-definition DECT at 90 and 150 Sn kVp and fast, low-dose, long-pitch CT for imaging COVID-19.

Pitch factor: Radiation dose reduction in CT can be achieved by altering scanning parameters, such as mAs, kVp, collimation, and pitch value [35,36]. The use of an ultralong pitch is the main feature of the LDCT protocol, and using a long pitch value prevents motion artifacts, thus improving image quality and reducing patient dose [40,41,42,43,44,45,46,47,48,49]. As demonstrated in this study, ULD, in particular, has been accompanied by a substantial increase in the pitch factor compared to the STD chest protocol [41,42].

Iterative reconstruction (IR) algorithms: Lowering kVp and mAs to lower the dose, as is needed in LDCT for imaging COVID-19 patients, causes more noise, which lowers the quality of the image and makes it necessary to use iterative reconstruction (IR) algorithms. IR algorithms have the potential for radiation dose optimization by lowering image noise [45]. IR algorithms have been used instead of filtered backprojection (FBP) reconstruction algorithms. Equipment vendors have used different models of IR algorithms with particular characteristics: CT scanners built by Siemens use ADMIRE IR algorithms, whereas GE Healthcare uses ASIR [40,41,43]. The use of IR is vital for eliminating increased image noise and artefacts because of the lowered tube current in low-dose CT. According to Gordon et al. [58], IR algorithm use resulted in a 52% reduction in noise compared to FBP. This is obvious in the current survey: relatively high effective doses were required when filtered FBP was used in the study by Tabatabaei et al. [44] and Li et al. [45] compared with other studies in which IR algorithms were used. A significant dose reduction was achieved when TCM was combined with IR algorithms.

### 3.3. Radiation Dose Considerations

This review addresses the differences in cumulative radiation dose exposure due to the successive use of ionizing radiation in different chest CT protocols for imaging COVID-19 pneumonia. Radiation doses are summarized in terms of *CTDI_vol_* and ED. *CTDI_vol_* quantifies the scanner output and improves equipment performance following the use of vendor-related optimization measures [59,60]. ED refers to the total radiation risk to the patient and is suitable for comparing different imaging modalities and techniques concerning the radiation burden [61]. Based on our previous dose surveys in Sudan, patients received effective dose values in the range of 4.6–5.2 mSv during routine CT examinations [62,63].

During COVID-19 pneumonia follow-up, a patient may undergo 6–8 chest CT scans, resulting in a significant cumulative dose. Zhou et al. [46] surveyed patient doses in 550 COVID-19 patients who underwent chest CT, noting a cumulative ED of 19.07 mSv for patients undergoing multiple CT examinations during the acute period of the COVID-19 pandemic.

Several studies comparing the STD chest CT protocol with the LDCT or ULD CT protocol used to image COVID-19 patients have been conducted. Table 3 summarizes the patient dose metric values in the STD, LDCT, and ULD chest CT protocols used for imaging COVID-19 patients. Table 4 provides information about the effective dose calculation.

**Figure 2:**
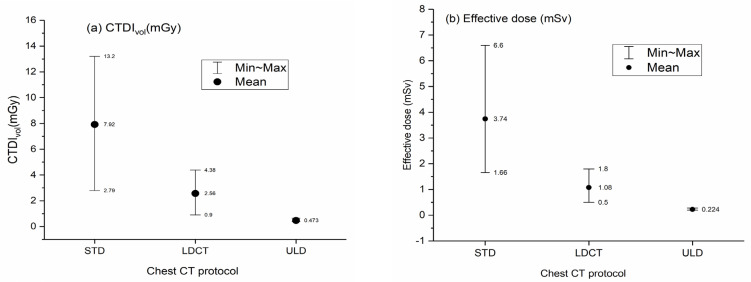
Boxplot presentation of LDCT and STD chest CT radiation dose distribution.

**Figure 3:**
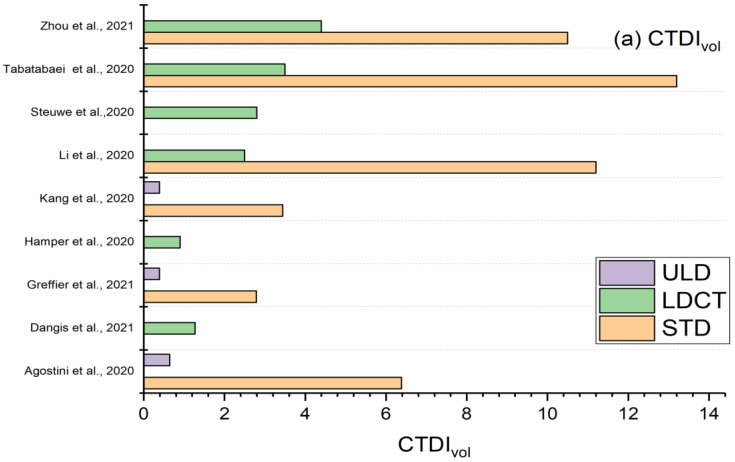

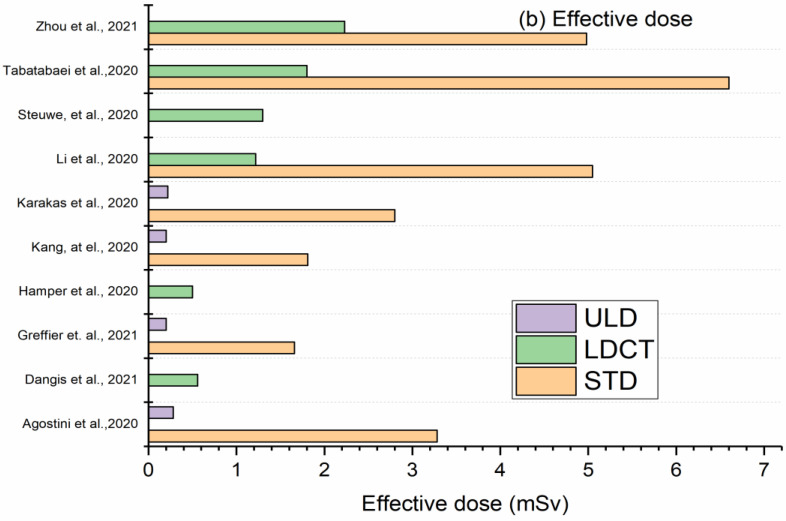
Bar chart comparison of LDCT and ULD vs. STD chest CT for imaging COVID-19; Agostini et al., 2020 [40]; Greffier et al., 2021 [41]; Kang et al., 2020 [42]; Karakaş et al., 2020 [43]; Tabatabaei et al., 2020 [44]; Li et al., 2020 [45]; Zhou et al. [46]; Dangis et al., 2020 [47]; Hamper et al.,2020 [48]; and Steuwe, et al., 2020 [49].

Figure 2 presents the aggregate results for both groups’ *CTDI_vol_* and ED using boxplots. Figure 3 presents a bar chart comparing the LDCT and ULD with STD protocols. 

The CTDIvol values ranged from 2.79–13.2 mGy in STD, 0.9–4.4 mGy in LDCT, and 0.20–0.28 mGy in ULD chest CT protocols. ED values ranged from 1.7–6.6 mSv in STD, 0.5–0.8 mSv in LDCT, and 0.39–0.64 mSv in ULD chest CT protocols. In this study, using the ULD chest CT protocol led to a dose reduction factor of 8–13, while using the LDCT chest protocol led to a dose reduction factor of 2–4 [40,41,42,43,44,45,46,47,48]. This survey revealed two ways to achieve dose reduction in CT scans [50]. This includes defining the target image quality that is adequate for a specific diagnostic task and achieving dose reduction by reducing the noise level using IR to improve the image quality. These results were obtained using various dose-reduction techniques, as discussed in the following sections.

In summary, Agostini et al. [40] reported an ED of 3.28 mSv in STD compared to 0.28 mSv in ULD protocols, resulting in a dose reduction of 11 times. The IR algorithm ADMIRE was used in conjunction with a 90/150Sn DSCT scanner. In another study, Greffier et al. [41] performed LDCT using a Siemens EDGE/Somatom CT scanner, 100 kVp, 10 mA, 1.7 pitch, and the ADMIRE 4 IR algorithm. Their results revealed a dose reduction by a factor of 8. Karakaş et al. reported ED values of 0.22 mSv in ULD as opposed to ED values of 0.28 mSv, indicating a 13-fold dosage decrease [43]. They used the GE Healthcare Optima 660 SE with 80 kVp and 10 mA ref, as well as the ASIR algorithm. In the ULD chest CT protocol, a significantly low tube current was used in conjunction with IR to preserve image quality. The dose was reduced by approximately 10 times that of the standard CT, comparable to the corresponding radiographic procedures.

Tabatabaei et al. [44] reported an ED of 6 mSv in STD protocols compared with 1.80 mSv in LDCT protocols. This means that the dose was reduced by a factor of 4. Similarly, Li et al. [45] reported ED values of 5.05 mSv in STD compared with 1.22 mSv in LDCT, achieving a dose reduction by a factor of 4. The dose reduction in ULD was almost double that achieved using LDCT. Owing to the degradation of image qualities causing difficult changes in kV and mAs, the ULD includes more technical features to comprehend the expected degradation in image quality.

Owing to the high dose reduction, ULD chest CT is inevitably associated with increased noise and artifacts, which necessitates the use of IR algorithms to compensate for the loss of image quality.

### 3.4. Image Quality Issues

Based on the results of the literature survey, the application of LDCT and ULD chest CT protocols for imaging COVID-19 patients is inevitably accompanied by concerns about image quality. The authors identified two sources that lead to lower image quality: COVID-19 patients are coughing, causing motion artefacts, while a lower radiation dose causes an increase in noise, both of which cause degradation in image quality, which is addressed by using LDCT with a high speed and ultra-long pitch.

In the surveyed literature, the authors evaluated image quality using objective and subjective methods [40,41,48,49], only objective methods [42], only subjective methods [43,45,47], and articles that did not include image quality evaluations [44,46]. Objective image quality evaluations were mainly based on contrast-to-noise (CNR) and signal-to-noise ratio (SNR) measurements [49], whereas subjective image quality evaluations were based on visual image quality ratings based on the radiologist’s perceptions.

Most authors have reported that ULD and ULD protocols are achievable with acceptable image quality. Agostini et al. [40] performed image quality evaluations and reported significant differences in SNR and CNR for several anatomical structures using the STD, LDCT, and ULD protocols. LDCT and ULD based on HD-DECT showed comparable diagnostic performance as well as a substantial reduction in motion artefacts. This is attributed to the use of an energy spectrum tin filter (100Sn kVp), which results in reduced image noise and radiation dose. Similarly, Kang et al. [42] used a tin filter (Sn100 kVp) and reported a significant reduction in the effective dose without a significant reduction in the image quality. Li et al. [45] performed subjective evaluations of image quality. The radiation dose was significantly reduced at acceptable image quality when using CareDose 4D along with Karl 3D IR in LDCT. Similar results were reported by Dangis et al. [47], who conducted subjective image evaluations demonstrating that LDCT at the sub-millisievert dose level permits imaging of suspected COVID-19. Hamper et al. [48] determined the CNR and SNR image quality parameters, demonstrating LDCT imaging with a radiation dose in the submillisievert range without sacrificing the quality of the diagnostic information.

However, few authors have expressed concerns when applying the LDCT and ULD chest CT protocols for imaging COVID-19 patients. According to Agostini et al. [40], the ULD chest protocol cannot be used to image overweight patients because the amount of radiation is insufficient to penetrate the body, resulting in poor image quality. This can be remedied by boosting the tube’s voltage and current using a CT scanner with tin filters that alter the spectrum’s shape or DECT.

Additionally, Karakaş et al. [43] carried out an objective image quality assessment, which shows a significant difference in the performance of SDT and LDCT. According to this study, LDCT may not be suitable for the initial imaging of suspected COVID-19 patients due to the requirement for higher sensitivity, but it is recommended for the follow-up of COVID-19 patients. In contrast, Steuwe et al. [49] reported that noise and CNR were significantly superior to ULD than STD and concluded that ULD might not be suitable for the long-term follow-up of viral pneumonia. The results demonstrated that, for lung imaging, there was a decrease in noise and an increased CNR with the ULD compared to the STD. These changes were related to an increase in the iterative level and the use of a softer reconstruction kernel.

The main limitation of this study is that we did not present quantitative image quality parameters. This is because there was no unified set of image quality parameters measured across all centers. However, we have summarized the major findings and recommendations of the reviewed literature, which emphasize the importance of image quality evaluation in conjunction with radiation dose measurement in the application of LDCT and ULD protocols.

## 4. Summary

This review summarizes the technical features of current low-dose CT (LDCT) protocols compared with standard chest CT protocols, focusing on radiation dose optimization challenges. During the acute phase of COVID-19 pneumonia, chest CT is used repeatedly to monitor the patients. During this time, a patient can undergo 6–8 CT scans, resulting in a significant cumulative radiation dose. In these cases, the use of standard CT for imaging COVID-19 results in a significantly effective dose, which is known to increase the probability of carcinogenesis. Overall, this has led to concerns regarding the use of ionizing radiation for COVID-19 pneumonia imaging. To address these concerns over radiation risk, CT equipment vendors and professionals have adopted several dose reduction techniques and optimization tools. Most patients with COVID-19 require fast scanning and higher pitch values owing to the shortness of breath or coughing. The major component of the LDCT protocol is the incorporation of dose reduction through both mA modulation and automatic tube voltage selection features, which are available in certain CT scanners. Dose reduction is often achieved by shaping using a tin filter at kVp and dual-energy CT (DECT). However, radiation dose reduction results in image artifacts that degrade image quality and necessitate the use of IR to preserve image quality.

The reviewed literature affirmed that the cumulative effective dose of multiple LDCTs may be less than or equivalent to that of the STD protocol. Therefore, LDCT can substitute for the STD protocol for imaging COVID-19 pneumonia with acceptable image quality.

## Figures and Tables

**Table 1:** Summary of the surveyed studies, detailing the objectives of each study, the subject and equipment used, and the major findings.

Study	Objectives	Study Population	Major Findings
Italy: Agostini et al. [40]	To study the feasibility of a ULD with fast, long-pitch, dual-source scanning	10 adult COVID-positive patients aged >18 years (average age: 53 years)	Reduced radiation dose with acceptable image quality was obtained using ULD with 100 Sn kV spectral shaping, dual-source, and ultra-long pitch.
France: Greffier et al. [41]	To compare the diagnostic performance of LDCT and STD using objective and subjective image quality measures	380 patients (M:F = 195:185) with a mean age of 66.3 ± 18.7 years; 97 CTs showed viral pneumonia	ULD is less effective than STD CT for examining the interstitial space and is not recommended for follow-up of these diseases.
China: Kang and Zhou [42]	To minimize radiation dose during chest CT imaging of COVID-19 using an LDCT protocol	An LDCT protocol was implemented using energy spectrum tin-filtering technology (Sn100 low-dose protocol)	The LDCT protocol resulted in a radiation dose reduction from 1/8 to 1/9 of the STD without significant loss of image quality.
Turkey: Karakaş et al. [43]	To use LDCT for the initial scanning of suspected COVID-positive patients	740 patients aged 44 ± 17 years (range: 18–97) who underwent both STD and LDCT	LDCT may not be suitable for scanning suspected COVID-19 patients where sensitivity is important, but it is preferred during follow-up because of its specificity.
Tabatabaei et al. [44]	To compare LDCT (30 mAs) with STD (150 mAs)	63 symptomatic patients (aged ≥ 50) with positive RT-PCR results for SARS-CoV-2 infection	Neither LDCT nor STD demonstrated a significant difference in the ability to detect COVID-19 pneumonia.
Li et al. [45]	To compare LDCT with STD for imaging COVID-19 using CareDose 4D and Karl’s 3D IR technology	56 patients (27 females); average age: 61.9 ± 13.7 years (range: 32–86)	The radiation dose is greatly reduced without significantly affecting image quality using LDCT with CareDose 4D combined with Karl 3D technology.
Zhou et al. [46]	To study radiation doses in chest CT used for imaging COVID-19 and assess the importance of LDCT	2119 non-contrast chest CTs of 550 patients across 92 hospitals; the patients were declared COVID-positive by RT-PCR	The authors recommend LDCT with rapid-scan, single-phase to reduce radiation dose and motion artefacts.
Dangis et al. [47]	To determine whether LDCT chest CT can be performed at a submillisievert dose level	192 patients with possible COVID-19 infection confirmed by RT-PCR test	Patients with symptoms lasting longer than 48 h can be assessed for COVID-19 infection using fast, accurate, and reproducible LDCT.
Hamper et al. [48]	To determine LDCT parameters sufficient for acceptable image quality	36 patients positive for SARS-CoV-2 (M/F = 27/9) were retrospectively included for chest CT scans.	LDCT gives radiation doses in the submillisievert range without compromising image quality.
Steuwe et al. [49]	To evaluate the image quality and radiation dose of an LDCT protocol and its diagnostic accuracy.	105 symptomatic patients with negative PCR results, aged 66.6 ± 16.7 years (range: 19–94 years).	The authors developed an LDCT protocol with high sensitivity to diagnose COVID-19 with sufficient image quality.

**Table 2:** Features of the LDCT, ULD, and STD chest CT protocols for imaging COVID-19 pneumonia.

CT Scanner Model/Made	Protocol	Tube Voltage	Reference TCTP/Modulation Software	IR Algorithm	Pitch	Reference
ULD vs. STD						
Dual-source CT scanners (DSCT), Siemens	STD	90/150 Sn kVp,	85 mAs Ref./CareDose 4D; 0.25 s	ADMIRE	1.1	Agostini et al. [40]
	ULD	100 Sn kVp,	180 mAs Ref./ CareDose 4D	ADMIRE	3.0	
Somatom EDGE/Siemens	STD	120 kVp	61 mAs/ CareDose 4D	ADMIRE 3	1.2	Greffier et al. [41]
	ULD	100 kVp	10 mA	ADMIRE 4	1.7	
Dose survey	STD	100 kVp,	85/62 mAs; 0.5 s	**	**	Kang and Zhou [42]
	ULD	100 Sn kVp,	112/96 mAs; 0.25s	**	**	
Optima 660 SE, GE Healthcare	STD	120 kVp	300 mA	ASIR	**	Karakaş et al. [43]
	LDCT	80 kVp	40 mA	ASIR	**	
LDCT vs. STD						
Alexion TSX-034A, Toshiba, Japan)	STD	120 kVp, fixed	150 mAs	**	**	Tabatabaei et al. [44]
	LDCT	120 kVp/fixed	30 mAs	**	**	
40-row uCT530 (United Imaging, Inc., China).	STD	120 kVp	130 mAs Ref./CareDose 4D	FBP	1.07	Li et al. [45]
	LDCT	120 kVp	30 mAs Ref./CareDose 4D	FBP	1.07	
Dose survey	LDCT	120 kVp	TCM	IR	0.7–1.5	Zhou et al. [46]
	LDCT	120 kVp	TCM	IR	0.7–1.5	
LDCT						
SOMATOM Definition AS 64-slice	LDCT	100 kVp	20 mAs/ CareDose 4D, 0.5 s	sinogram-affirmed	1.2	Dangis et al. [47]
GE Light-Speed Aquilion Prime	LDCT	100 kVp	10−120 mA	AIDR 3D standard	1.388	Hamper et al. [48]
Somatom Definition Edge/ Siemens	LDCT	100 kVp	60 mAs/ CareDose 4D	ADMIRE	0.6	Steuwe et al. [49]

TCTP: tube current-time product; ADMIRE: advanced modeled iterative reconstruction; ASIR: adaptive statistical iterative reconstruction; FBP: filtered back projection; ** missing information.

**Table 3:** Summary of radiation doses in the chest CT imaging of COVID-19 patients.

Country/Study (Sample Size)	Protocol	*CTDI_vol_* (mGy)	*DLP* (mGy cm)	Effective Dose (mSv)	Dose Reduction *
ULD vs. STD					
Italy: Agostini et al. [40] (*n* = 10)	STD	6.38 (3.9–7.5)	226.2 (176–322)	3.28 (2.6–4.7)	11
	ULD	0.64 (0.47–1.12)	19.5 (17.5–29.0)	0.28 (0.3–0.4)	
France: Greffier et al. [41] (*n* = 380)	STD	2.79 (2.2–3.5)	118.6 (92–156)	1.66 (1.29–2.18)	8
	ULD	0.39 (0.39–0.40)	14.2 (13.1–15.4)	0.20 (0.18–0.22)	
China: Kang and Zhou [42]	STD	3.44	129.1	1.81	9
	ULD	0.39	14.5	0.20	
Turkey: Karakaş et al. [43] (*n* = 740)	STD	Not reported	190 (98–494)	2.8 (1.4–6.9)	13
	ULD	Not reported	15.59 (12–32)	0.22 (0.16–0.45)	
LDCT vs. STD					
Iran: Tabatabaei et al. [44] (*n* = 20)	STD	13.2 ± 2.5	412.8 ± 91.7	6.60 ± 1.47	4
	LDCT	3.5 ± 0.8	112.2 ± 26.6	1.80 ± 0.42	
China: Li et al. [45]	STD	11.21 ± 1.50	360.50 ± 52.99	5.05 ± 0.74	4
	LDCT	2.53 ± 0.27	87.25 ± 10.21	1.22 ± 0.14	
China: Zhou et al. [46] (*n* = 2119)	STD	10.5 (0.6–33.8)	355 (6.8–1098)	4.98 (0.1–15.2)	2
	LDCT	4.382	159.43	2.23	
LDCT					
Belgium: Dangis et al. [47] (*n* = 192)	LDCT	1.27 ± 0.59	39.9 ± 17.8	0.56 ± 0.25	**
Germany: Hamper et al. [48] (*n* = 36)	LDCT	0.9 ±0.3	35 ± 10.2	0.5 ± 0.2	**
Germany: Steuwe et al. [49] (*n* = 105)	LDCT	2.8 ± 0.9	89.3 ± 27.7	1.3 ± 0.4	**

* Dose reduction = ED(STD)/ED(LDCT); ** missing information

**Table 4:** Methods used for effective dose calculation.

Study	ED Calculation Method	Remarks
Italy: Agostini et al. [40]	0.0145 mSv/mGy·cm	Dose conversion coefficient
France: Greffier et al. [41]	0.0144 mSv/mGy·cm	Dose conversion coefficient
China: Kang and Zhou [42]	0.014 mSv/mGy·cm	Dose conversion coefficient
Turkey: Karakaş et al. [43]	DoseWatch	Dose-tracking software
Iran: Tabatabaei et al. [44]	0.016 mSv/mGy·cm	Dose conversion coefficient
China: Li et al. [45]	0.014 mSv/mGy·cm	Dose conversion coefficient
China: Zhou et al. [46]	0.014 mSv/mGy·cm	Dose conversion coefficient
Belgium: Dangis et al. [47]	0.014 mSv/mGy·cm	Dose conversion coefficient
Germany: Hamper et al. [48]	CT-Expo 2.5^®^ software	Monte Carlo-based CT dosimetry software
Germany: Steuwe et al. [49]	0.014 mSv/mGy·cm	Dose conversion coefficient

## Data Availability

Data are available upon reasonable request from the corresponding author.
